# Roles of the *Drosophila* SK Channel (dSK) in Courtship Memory

**DOI:** 10.1371/journal.pone.0034665

**Published:** 2012-04-11

**Authors:** Ahmad N. Abou Tayoun, Claudio Pikielny, Patrick J. Dolph

**Affiliations:** 1 Department of Biology, Dartmouth College, Hanover, New Hampshire, United States of America; 2 Department of Genetics, Dartmouth Medical School, Hanover, New Hampshire, United States of America; University of Missouri, United States of America

## Abstract

A role for SK channels in synaptic plasticity has been very well-characterized. However, in the absence of simple genetic animal models, their role in behavioral memory remains elusive. Here, we take advantage of *Drosophila melanogaster* with its single *SK* gene (*dSK*) and well-established courtship memory assay to investigate the contribution of this channel to memory. Using two independent *dSK* alleles, a null mutation and a dominant negative subunit, we show that while dSK negatively regulates the acquisition of short-term memory 30 min after a short training session, it is required for normal long-term memory 24 h after extended training. These findings highlight important functions for dSK in courtship memory and suggest that SK channels can mediate multiple forms of behavioral plasticity.

## Introduction

Learning and memory are dependent on changes in synaptic strength between relevant neurons. Several molecular components, including ion channels, have been identified that contribute to synaptic strengthening in an experience-dependent manner [1] . The small conductance calcium-activated potassium (SK) channel couples changes in intracellular Ca^2+^ to membrane potential and therefore is an essential regulator of neuronal function. At the synapse, SK channels negatively regulate both neurotransmitter release and synaptic plasticity by forming negative feedback loops with the nearby Ca^2+^ sources. Consequently, blocking those channels with the bee venom toxin, apamin [2], enhances synaptic transmission and lowers the threshold for *N*-Methyl-D-aspartate receptor (NMDAR)-dependent long-term potentiation (LTP) [3], [4], [5], [6], a measure of synaptic strength.

Specific inhibition of SK channels using apamin provided strong evidence for their role in learning and memory processing. However, the behavioral role of SK channels remains controversial. Consistent with their role in synaptic plasticity, there are multiple reports that apamin treatment enhances learning and memory [4], [7], [8], [9]. Nonetheless, in some cases apamin-treated animals showed normal memory [10], [11], [12] while in others memory retention was severely disrupted [13], [14]. These discrepancies may be due to different experimental and behavioral paradigms utilized in each study and differential sensitivities to apamin exhibited by multiple SK subtypes [15]. To overcome these limitations, we decided to test the role of SK channels in behavioral memory using *Drosophila melanogaster* genetics and its well-established courtship memory assay [16].

Unlike mammals, the fruit fly contains a single highly conserved *SK* gene in its genome (*dSK*) [17], simplifying genetic analysis of SK function *in vivo*. Here, using a *dSK^−^* null fly [17], we show that dSK underlies at least two courtship memory traces. Compared to control flies, *dSK^−^* mutants exhibited enhanced short-term memory 30 min after a short training session. On the other hand, long-term memory was defective in mutants 24 h after extended training. We confirm these data using a dominant negative dSK subunit and the GAL4/UAS system [18] to inactivate dSK in different groups of central neurons. Our results suggest that dSK's functions in courtship memory are required in non-overlapping subsets of neurons, defined by the GH146- and 201y-GAL4 driver lines.

## Materials and Methods

### Fly Strains

The *dSK^−^* null allele was generated using *FLP-FRT*-mediated homologous recombination [17], [19]. The resulting *dSK^−^* allele was then outcrossed to wild type Canton-S (*w^+^*). Both *w^+^* (wild type) and *w^+^dSK^−^* flies were further outcrossed to FM7a background (isogenic on the second and third chromosomes) for 4 generations to generate two isogenic lines differing only at the dSK locus. Similarly, all transgenic lines were crossed into a Canton S (*w^+^*) background. To express dSK dominant negative subunit [17] in desired neurons, we crossed *UAS-SKDNmyc* homozygous females to either *201y-GAL4* (Bloomington stock center) or *GH146-GAL4* (gift from Gilles Laurent) homozygous males, respectively, or to wild-type males to create the UAS heterozygous effector control. Both GAL4 lines were crossed to wild-type females to create heterozygous driver controls.

### Behavioral Analysis

Flies were raised on standard cornmeal and molasses medium at 25°C and 60–70% humidity in a 12-h light-dark cycle. Flies were collected at eclosion and aged for 6–7 d. Males were aged individually while females were aged in groups of 15 per food vial. *W^1118^* females were pre-mated by pairing 10 females with 15 Canton-S males (>5 d old) for ∼18 h and then retrieved 2–3 h before being used for training. Males were assayed for courtship conditioning using the repeat training test [20], [21], [22].

For the short-term memory training, individual males were placed in small or large chambers either with (trained) or without (naïve) a single pre-mated female for 1.5 h. For the long-term memory experiments, extended training is achieved by conditioning in large food chambers for 5 h [23]. After training (or sham training), each male was transferred to a fresh food vial and kept in isolation at 25°C until testing. During the 10 minute test period, both naïve and trained males were individually paired with a newly mated female in a solid Plexiglas (7 mm diameter×7 mm deep) recording chambers where they were videotaped. Subsequently, courtship index for each male was calculated as the percentage of time he spent engaging in courtship during the 10 minute test period. The learning index (LI) was calculated as the percentage reduction in mean courtship activity of trained males relative to that of naïve males [22]:
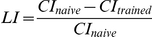
To measure the courtship suppression during training (see below), individual males were placed in a solid Plexiglas chamber (7 mm diameter×7 mm deep) with a single pre-mated female, and their behavior recorded for 30 minutes after which courtship was scored blind.

All 1411 assays were scored for the courtship index by the same experimenter while blind to both genotype and experimental condition.

### Statistical Analysis

Statistical significance of the difference between naïve and trained flies within each group was assessed using a 2-sample t-test. A paired t-test was used to calculate significance in courtship suppression during training for the same group (see below). For learning indices, statistical significance was calculated using a custom script in STATA to perform permutation tests as described before [21], [22]. This analysis repeatedly randomizes the observed courtship indices creating a universe of hypothetical LIs allowing us to calculate the probability of getting the real experimental LI by chance. Within each experimental condition, the entire set of naïve and trained courtship indices were pooled and randomly assorted into simulated naïve and trained sets of the same size as in the original data [21], [22]. A LI_P_ was calculated for each of 100,000 randomly permuted datasets, and P values were estimated as the fraction for which LI_P_>LI (to test H_0_: LI = 0) or |LI_P_|>|LI−LI_0_) (to test H_0_: LI = LI_0_). As the distributions of the LIs differ between groups, we chose to use this distribution free randomization test, as in previous similar analyses [21], [22].

## Results

We assayed learning and memory in *dSK^−^* flies using courtship conditioning, where a male fly shows an experience-dependent courtship suppression towards females following prior exposure to a mated unreceptive female [16]. In this paradigm, individual male flies are singly placed in a training chamber with (trained) or without (naïve) single, previously mated wild-type female. After isolation for a rest period, trained and naïve males are singly tested for courtship suppression or memory by subsequent pairing with a second mated female (repeat training assay) [24]. Here, we tested the ability of *dSK^−^* flies to form short-term memory 30 min after a 1.5 h (short) training session and long-term memory 24 h after a 5 h (extended) training session. Unlike the mushroom body (MB)-dependent memory formed by extended training, in the former paradigm earlier memory phase (up to 30 minutes) is MB-independent while subsequent memory requires this structure [23].

### A mutation in *dSK* results in increased acquisition of short-term memory

**Figure 1:**
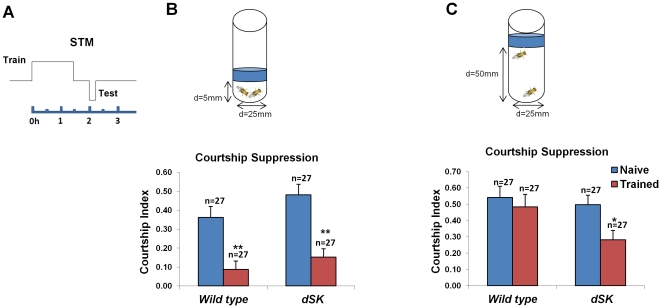
A mutation in *dSK* results in increased acquisition of short-term memory 30 min after 1.5 h ‘short’ training session. Short-term memory (STM) paradigm. During training period (1.5 h), male flies are individually placed in small chambers with (trained) or without (naïve) mated females in small (B) or large (C) training chambers. After a 30 min rest period in isolation, each male was tested by pairing with a mated female for 10 min. (A) Courtship indices of naïve and trained males for each group were measured during testing with mated females 30 min after STM training (A) in small chambers. Note the significant suppression in courtship levels of trained males relative to that of the naïve flies within each group. Mean+SEM is shown. (**P<0.01, t test). (B) Same as in (B) except that training is limited by increasing chamber size as shown. Only trained *dSK^−^* flies exhibit robust courtship suppression. Mean+SEM is shown. (*P<0.05, t test).

To assay short-term memory, conditioning is carried out in a small chamber to achieve a short training session where the male would be in almost constant contact with the mated female [16]. Such conditions have been shown to induce short-term courtship suppression lasting few hours. Our wild-type male flies showed a strong suppression of courtship when tested 30 minutes after a 1.5 hour training session in small chambers (Figure 1A and 1B). We also observed identical short-term courtship suppression in *dSK^−^* flies (Figure 1B).

While these results suggested that *dSK^−^* mutants have normal learning and memory, we reasoned that any phenotype may have been masked by our training paradigm in small chambers, which may represent a ‘high-intensity’ training protocol. To improve the sensitivity of our assay, we conditioned flies for the same period of time (1.5 h) while increasing the training chamber size (Figure 1C). Under such conditions males are expected to have less interaction with mated females [24] and, therefore, a relatively weaker training session. Indeed, increased chamber size drastically affected memory levels in control flies, which showed no significant courtship suppression 30 min post-training (Figure 1C). Strikingly, under these training conditions, only *dSK^−^* flies exhibited a highly significant 30 min memory level (Figure 1C) suggesting that mutants had a lower threshold for training acquisition and implying that the normal function of dSK is in negatively regulating short-term memory acquisition. Mutant naïve males exhibited courtship levels towards mated females similar to that of control flies (Figure 1B and 1C), thus ruling out any sensorimotor defects relevant to courtship behavior towards mated females.

### A mutation in *dSK* results in decreased long-term memory

**Figure 2:**
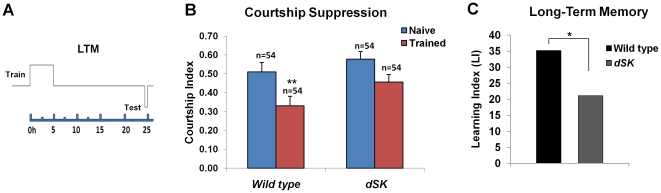
A mutation in *dSK* results in decreased long-term memory 24 h after 5 h ‘extended’ training session. (A) Long-term memory (LTM) paradigm. During training period (5 h), male flies are individually placed with (trained) or without (naïve) mated females in food chambers. After a 24 h rest period in isolation, each male was tested by pairing with a mated female for 10 min. (B) Courtship indices of naïve and trained males for each group were measured during testing period 24 h post-training. Unlike trained wild-type males (**P<0.01, t test), trained *dSK^−^* flies exhibit weak courtship suppression (P>0.05). Mean+SEM is shown. (C) Learning indices (LIs) of wild-type and *dSK^−^* males were calculated from courtship indices of naïve and trained males as described before [22] (also see text). Note that 24 h memory level (LI) is significantly lower for *dSK^−^* males compared to wild type. Mean LI is shown. *P<0.05 for H_0_: LI = LI_wild type_.

To establish a long-term courtship memory, a longer 5 h training session is carried out in food chambers [23]. Unlike short-term memory, this memory form is likely protein synthesis dependent [22] and lasts for at least 24 hours [23]. We therefore evaluated whether dSK is required for long-lasting memory produced by this paradigm (Figure 2A). Similar to previous reports [22], [23], our control flies exhibited courtship suppression when tested 24 hours post-training (Figure 2B). Strikingly, *dSK^−^* flies exhibited unexpectedly weak courtship suppression at 24 hours post-training. To compare long-term memory scores between control and mutant flies, we estimated the learning index for each group as described before [22] by calculating the percentage reduction in mean courtship activity of trained males relative to that of naïve males. Compared to control flies (LI = 35.2), *dSK^−^* flies had significantly lower memory levels (LI = 21.1, P = 0.017 for H_0_: LI = LI_wild type_, Figure 2C). Therefore, our data suggest that while the dSK channel activity negatively regulates the acquisition of short-term memory, it is also required for the formation of long-term memory.

### Effects of dSK inactivation on short-term memory acquisition

Similar to mammalian SK channels, dSK is broadly expressed in the adult fly brain [17] suggesting that it is present in most, if not all, brain neurons. In an effort to confirm our findings we chose to generate flies where dSK is inactivated in non-overlapping neurons defined by the GH146- and 201y-GAL4 driver lines which collectively drive expression in several neurons including the projection neurons (which show highest dSK expression, see below), the anterior paired lateral neurons (APLs), the MB neurons, and the lateral protocerebrum [25], [26], [27], [28]. All these neurons have well-established roles in olfactory and courtship memory [22], [27], [29], [30], [31], [32].We exploited the fact that a functional dSK channel is a tetramer and therefore generated a myc-tagged dominant negative dSK subunit (*UAS-dSKDNmyc*) by mutating the K^+^ pore ‘GYG’ into ‘AAA’, a mutation that has been used to inactivate several K^+^ channels in different systems [33], [34], [35]. When expressed in wild-type background, this subunit completely abolished the dSK current observed in R1–R6 photoreceptors and phenocopied enhanced *in vivo* kinetics observed in *dSK^−^* null photoreceptors [17]. We expressed this dSK dominant negative subunit (dSKDN) in either GH146- or 201y-positive neurons and asked whether dSK channel inactivation in either set of neurons underlies the short-term memory enhancement observed in *dSK^−^* flies after short training in large chambers.

**Figure 3:**
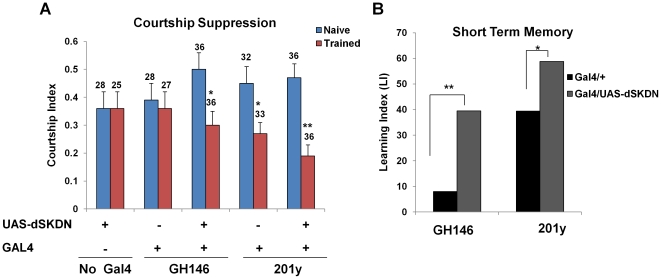
Effects of dSK inactivation on 30 min courtship memory acquisition. (A) Courtship indices of naïve and trained males for each group were measured during testing with mated females 30 min after STM training in large chambers. Mean+SEM is shown. (*P<0.05, **P<0.01, t test). (B) Learning indices (LIs) were calculated from courtship indices of naïve and trained males within each group as described before [22] (also see text). Note the significant 30 min memory enhancement when dSKDN is expressed either sets of neurons. Mean LI is shown. *P<0.05 for H_0_: LI = LI_201y-GAL4_, **P<0.01 for H_0_: LI = LI_GH146-GAL4_.

Similar to wild-type flies (Figure 1), our results show that neither the UAS/+ effector nor GH146/+ driver control groups had significant courtship suppression 30 min after training (Figure 3A). On the other hand, flies expressing dSKDN in GH146 positive neurons interestingly showed enhanced short-term memory acquisition (Figure 3A). Similarly, dSK inactivation in 201y positive neurons resulted in a robust courtship suppression. However, the 201y/+ driver control group also showed significant suppression when tested 30 min after training (Figure 3A). To control for nonspecific effects of individual GAL4 drivers, we compared learning indices of males expressing dominant negative dSK in specific brain regions to that of the corresponding GAL4 driver control. Interestingly, dSK inactivation in 201y positive neurons yielded a modest (only∼50%) increase in short-term memory when compared to its GAL4 driver control (P<0.024 for H_0_: LI = LI_201y-GAL4_, Figure 3B), while a five-fold enhancement was observed when dSKDN was expressed in the GH146 neurons (P<0.0005 for H_0_: LI = LI_GH146-GAL4_ 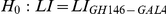, Figure 3B). This confirms the effects observed in *dSK^−^* flies and suggests that different cells mostly within the GH146 and likely 201y positive neurons are required for dSK-mediated 30 min courtship memory.

It is worth noting that naïve males carrying either of the drivers or effector transgenes alone or combined show normal courtship levels towards mated females ruling out any sensorimotor defects (Figure 3A).

### Effects of dSK inactivation on long-term memory

**Figure 4:**
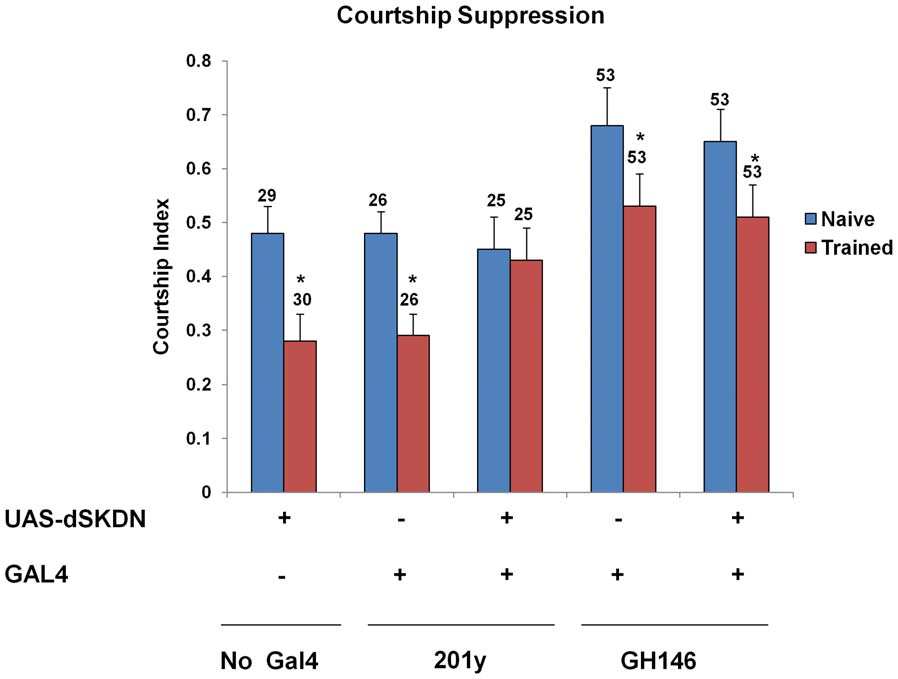
Effects of dSK inactivation on 24 h courtship memory formation. Courtship indices of naïve and trained males for each group were measured during testing with mated females 24 h after LTM training. Note that only flies expressing dSKDN in 201y-positive neurons do not show training-induced courtship suppression and therefore are severely impaired in 24 h courtship memory. Mean+SEM is shown. (*P<0.05, t test).

We next tested the effects of dSK inactivation in either GH146 or 201y neurons on 24 h, long-term memory assayed after a 5 h training session (Figure 2A). As expected, all driver and effector control groups exhibited significant courtship suppression when tested 24 h post-training (Figure 4). Although dSK inactivation in GH146 positive neurons yielded normal courtship suppression, a severe long-term memory defect was observed only when dSK dominant negative subunit was expressed using the 201y driver (Figure 4) suggesting that the role of dSK in 24 h courtship memory is likely independent of GH146 neurons, instead involving, at least, those defined by the 201y driver.

### A mutation in *dSK* results in enhanced courtship suppression during training

**Figure 5:**
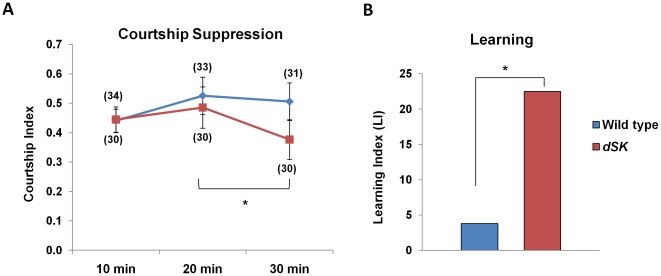
A mutation in *dSK* results in enhanced courtship suppression during training. (A) Courtship indices of wild-type and mutant males during individual pairing with mated females for 30 min. Courtship behavior was recorded and analyzed in 10 min bouts. Unlike controls, *dSK^−^* males exhibit a significant decrease in their courtship levels during the last bout (CI_30_) when compared to peak courtship activity (CI_20_). Data are Mean ± SEM. (*P<0.05, paired t test). (B) Learning index (LI) for each group was calculated as the percentage decrease in courtship activity during the last 10 min (CI_30_) relative to peak courtship levels (CI_20_). (*P<0.05 for H_0_: LI = 0).

In addition to subsequent courtship suppression, the courtship conditioning paradigm has been shown to have a distinct behavioral output during the training period where the male modifies his courtship of the mated female. This behavior is independent of the subsequent memory [36], [37] and requires a distinct neuronal circuit that involves CaMKII activity in the antennal lobes and parts of the lateral protocerebrum [27]. To test the effect of the *dSK* mutation on this behavior, we monitored the first 30 minutes during training in small recording chambers. Both control and mutant males showed similar courtship levels during the first 10 minutes that peaked during the next 10 minutes, suggesting that *dSK^−^* flies have intact sensory modalities underlying courtship conditioning. While control flies continued to court at the same level, *dSK^−^* males showed a significant decrease in their courtship activity in the last 10 minutes (Figure 5). This enhancement suggests that dSK negatively regulates courtship suppression during training and further highlights the importance of this channel in behavioral plasticity.

## Discussion

Using the *dSK^−^* null allele and a dominant negative dSK subunit, our data highlight important functions for dSK in *Drosophila* courtship memory thus confirming a critical role for this channel in behavioral memory. Our findings suggest that short and extended training paradigms induce two independent memory traces in which dSK plays different roles. While it negatively regulates 30 min memory acquisition, dSK is required for 24 h memory formation. This study is the first to show that dSK has opposite functions in short- and long-term memory likely involving two non-overlapping sets of neurons. Although we can speculate about the identity of relevant neurons, future experiments using several specific GAL4 drivers would be indispensable for further refining the cellular requirements for dSK functions in courtship memory.

### dSK down regulates short-term courtship memory

Collectively, our behavioral data from *dSK^−^* flies and flies expressing a dominant negative dSK subunit in GH146- and likely 201y-positive neurons suggest that a short training session induces a 30 min memory phase that is negativey regulated by dSK. These observations are intriguing in light of pharmacological data that suggest a negative role for mammalian SK2 channel in long-term potentiation [3], [5].

**Figure 6:**
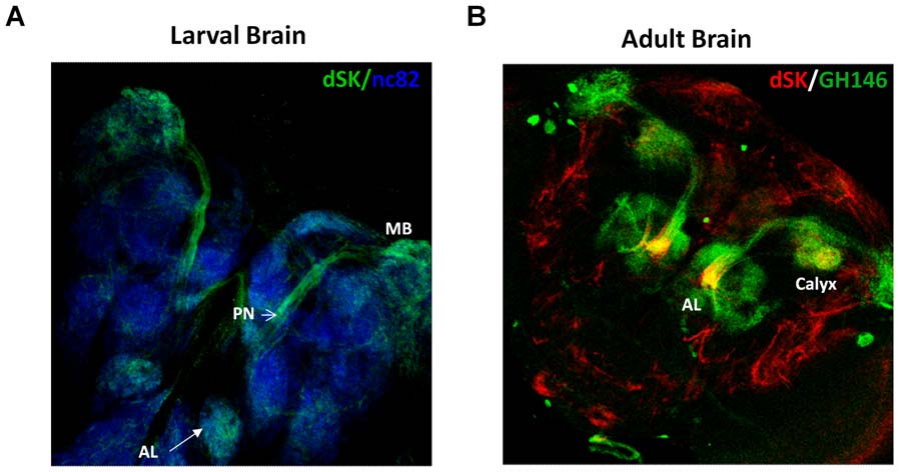
dSK expression in larval and adult brains. (A) A single confocal section through a WT larval brain stained with dSK antibody (green) and pre-synaptic marker nc82 (blue). dSK is highly expressed in PNs connecting ALs and MB calyx. (B) A confocal section through a *GH146-GAL4/+;UAS-mCD8GFP/+* stained with anti-dSK antibody (red). Notice dSK expression in PNs both in ALs and MB calyx.

The different effects obtained by ectopic expression of dominant negative dSK subunit under control of the GH146 and 201y-GAL4 drivers could be due to differences in expression levels, the different number of cells in which each driver is expressed or to expression in different brain regions. The GH146 driver line labels 83 projection neurons (PNs) (Figure 6), the anterior paired lateral (APL) neurons, 1 to 2 cell bodies (in addition to APL) near the lateral protocerebrum, in addition to a subset of neurons in the optic lobe [25], [26]. The 201y positive neurons include mainly the γ-lobe and the core of the α- and β-lobes of the MB. In addition, this driver also expresses in cells of the pars intercerebralis, lateral protocerebrum, and the subesophegeal ganglion [27], [28]. Both the PNs and APL neurons have been shown to mediate early memory formation using classical olfactory conditioning [29], [30], [31] and courtship conditioning [27]. Mushroom body – including the α-, β-, and γ- lobe – neurons defined by the 201y-GAL4 driver line have a well-established role in early memory using classical olfactory [32], [38], [39] and courtship conditioning [22], [27]. Hence, dSK function in either set or all of those neurons might be essential for early courtship memory. Given that dSK is expressed at highest levels in projection neurons (Figure 6), it is attractive to speculate that dSK inactivation in those neurons might underlie the increased acquisition of early memory, a possibility that could be tested in future experiments.

### dSK stimulates long-term courtship memory

Our behavioral data using *dSK^−^* flies and targeted expression of dominant negative dSK subunit in 201y-positive neurons also suggest that extended training induces a dSK-dependent 24 h, long-term memory trace. Our results strongly suggest that, unlike its negative regulation of early memory acquisition, dSK stimulates 24 h memory formation.

Interestingly, this memory phase showed no requirement for dSK function in GH146-positive neurons and may therefore be independent of the dSK-mediated 30 min memory formed in those neurons. Indeed, it has been reported that ‘short’ and ‘extended’ training paradigms lead to independent early memory traces in distinct neurons [23]. A testable hypothesis based on our findings is that while a short training session induces an early memory phase that is negatively regulated by dSK in GH146 neurons and in 201y neurons , extended training induces a 24 h memory that is stimulated by dSK in 201y neurons. Within the 201y neurons, early and late dSK-mediated memory forms could be dependent on different sets of neurons or on different molecular mechanisms within the same neurons.

In conclusion, this work highlights the differential contribution of dSK to two distinct courtship memory phases likely involving non-overlapping cells, and provides a tractable genetic system for the future dissection of the cellular and molecular mechanisms underlying the roles of dSK in short- and and long-term memory.
